# Increased Bone Formation and Accelerated Bone Mass Accrual in a Man Presenting with Diffuse Osteosclerosis/High Bone Mass Phenotype and Adenocarcinoma of Unknown Primary

**DOI:** 10.1002/jbm4.10734

**Published:** 2023-06-14

**Authors:** Terrence H. Diamond, Carl Bryant, Richard Quinn, Sindhu T. Mohanty, Fiona Bonar, Paul A. Baldock, Michelle M. McDonald

**Affiliations:** ^1^ Department of Endocrinology St George Hospital Campus, University of New South Wales Sydney Australia; ^2^ Department of Radiology St George Private Hospital Sydney Australia; ^3^ Department of Nuclear Medicine St George Private Hospital Sydney Australia; ^4^ Skeletal Diseases Program The Garvan Institute of Medical Research Darlinghurst Australia; ^5^ Department of Anatomical Pathology Douglass Hanly Moir Pathology and Royal Prince Alfred Hospital Sydney Australia; ^6^ St Vincent's Clinical Campus School of Clinical Medicine, University of New South Wales Kensington Australia; ^7^ School of Medicine Science, Faculty of Medicine and Health The University of Sydney Sydney Australia

**Keywords:** BONE DENSITY, HIGH BONE MASS, OSTEOSCLEROSIS

## Abstract

A 71‐year‐old man was referred for evaluation of incidental generalized osteosclerosis. He was found to have a high bone mass (HBM) with an elevated lumbar spine bone mineral density (BMD) Z‐score of +5.3. Over an 18‐month period, his lumbar spine BMD measured by dual energy X‐ray absorptiometry (DXA) had increased by +64% from 1.09 to 1.79 g/cm^2^ and femoral neck by +21% from 0.83 to 1.01 g/cm^2^. Biochemical markers of bone turnover were markedly increased (serum propeptide of type 1 collagen and urine telopeptides greater than 10‐times normal). The high bone formation and increased skeletal calcium acquisition resulted in profound hypocalcemia (low serum calcium 1.88 mmol/L) and hypocalciuria (low urinary calcium <0.2 mmol/day). Positron emission tomography (PET) with 2‐deoxy‐2‐[fluorine‐18] fluoro‐D‐glucose (FDG) confirmed diffuse osteosclerosis without focal areas of abnormal FDG uptake in the skeleton or elsewhere to suggest either an underlying primary malignancy or metastatic disease. Bone biopsy showed markedly sclerotic woven and lamellar bone. The marrow space was devoid of typical bone cells and adipocytes and instead was filled by fibromyxoid stroma, infiltrated by small clusters of tumor cells. Bone histomorphometry and micro–computed tomography demonstrated an elevated trabecular bone volume and trabecular plate thickness. The bone disorder in this case is unique and raises the possibility of a new yet undefined novel anabolic paracrine factor (or factors) secreted by an adenocarcinoma of unknown primary that resulted in dramatic increases in BMD, HBM, and radiological osteosclerosis. The differential diagnosis and potential mechanisms responsible for the HBM are discussed. © 2023 The Authors. *JBMR Plus* published by Wiley Periodicals LLC on behalf of American Society for Bone and Mineral Research.

## Graphical abstract

Lumbar spine and femoral neck BMD showed marked increases in the 18 months prior to presentation with incidental osteosclerosis. Iliac crest biopsy reveals dense trabecular bone reflecting sclerosis at the time of presentation.

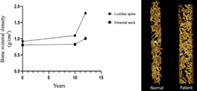

## Introduction

High bone mass (HBM) is defined as a bone mineral density (BMD) Z‐score ≥+2.0 and occurs with an incidence of three per 100,000 individuals.^(^ ^1^ ^)^ Thirty‐five percent of referrals are usually for suspicious malignant bone disease, and 22% of patients have an underlying medical disorder. Fifty percent are usually due to artifact elevations in BMD (Table 1).^(^ ^1^ ^)^ While the original definition of HBM is based on elevated dual‐energy X‐ray absorptiometry (DXA) Z‐scores, there are no data relating to dual‐energy quantitative computed tomography (QCT) Z‐scores, which are more accurate for quantitating “true” vertebral body cancellous BMD and eliminating artifact elevations.^(^ ^2^ ^)^

**Table 1:** Known Causes of High Bone Mass^(^ ^1^ ^)^

Artifactual elevations in BMD
Osteoarthritis, diffuse interstitial skeletal hyperostosis, ankylosing spondylitis
Vertebral fractures
Vascular calcification
Thalassaemia major, Gaucher's disease
Intra‐abdominal (renal calculi, gallbladder calculi, intestinal barium, silicone implants, surgical metalwork)
Laminectomy/fusion/vertebroplasty/kyphoplasty
Localized disorders
Paget's disease
Hypophosphatasia
Tumors—primary or secondary
Chronic osteomyelitis
Generalized disorders
Congenital
Decreased/absent bone resorption
*Osteopetrosis*
*Pycnodysostosis*
*Osteopoikilosis*
*Melorheostosis*
Increased bone formation
*Sclerosteosis/Van Buchem's*
*LRP5/LRP4 mutation*
*SMAD9 mutation*
Uncoupling bone formation/resorption
*Camurati Engelmann*
*Ghosal*
Acquired
Fluorosis
Renal osteodystrophy
Acromegaly
Hepatitis associated osteosclerosis
Mastocytosis
Hematological disorders
Malignancy
Anti‐osteoporotic therapies

HBM can be present at birth or be acquired. There are rare genetic disorders that affect osteoblast activity, resulting in an increase in bone formation via the Wnt/ß‐catenin signaling pathway, and are termed sclerosing bone dysplasias.^(^ ^3^, ^4^ ^)^ They comprise loss‐of‐function *SOST* mutations (Slereosteosis and Van Buchem's disease) or gain‐of‐function LRP4, LRP5, and LRP6 mutations. Other genetic disorders called osteopetrosis result from a decrease in osteoclast bone resorption.^(^ ^5^ ^)^ Acquired HBM is encountered with toxic fluoride or heavy metal exposure, mastocytosis,^(^ ^6^, ^7^, ^8^ ^)^ and as a tumor manifestation. HBM is commonly seen in prostate cancer^(^ ^9^ ^)^ but has also been reported to occur with breast and pancreas; mucinous adenocarcinoma of the gastrointestinal tract; transitional cell carcinoma; carcinoid tumor; and myeloma, lymphoma, and neuroblastoma.^(^ ^10^ ^)^

We report on an unusual case of acquired generalized osteosclerosis in a man who was found to have HBM with an elevated lumbar spine DXA Z‐score of +5.3 and a QCT Z‐score of +11.0 as a consequence of rapid bone mass accrual most likely from an adenocarcinoma of an unknown primary.

## Materials and Methods

### Case presentation

The patient was a 71‐year‐old man referred for incidental osteosclerosis noted on a recent abdominal CT scan. He had been previously well until 6 months prior to his presentation in 2016, when he developed abdominal discomfort, mild fatigue, and low‐grade back pain. He was investigated by gastroscopy and colonoscopy and diagnosed with chronic gastritis. On evaluation he appeared otherwise well, with ormal blood pressure 110/70 mm Hg, heart rate 76 beats per minute, and body mass index 24.6 kg/m^2^. There were no signs of tetany, bone deformities, exostoses, or torus palatinus, and his dentition was normal. There were several 1‐ to 3‐mm darkly pigmented verruca‐looking warts on the plantar surfaces of his hands and temple regions of his face. Cardiorespiratory and neurological examinations were normal. Abdomen was normal to palpation, there was no lymphadenopathy or hepatosplenomegaly, and his rectal examination revealed a prostate apparently devoid of lesions/nodularity on manual exploration. Systematic review confirmed long‐standing gastroesophageal reflux managed on esomeprazole and hypertension treated with amlodipine/valsartan combination. He was retired, living with his wife, a nonsmoker who rarely drank alcohol. He had no fracture history and had never been treated with bisphosphonates or bone anabolic agents. There was no previous history of exposure to heavy metals, fluoride, or herbal preparations and no intravenous drug use. There was no family history of osteosclerosis or bone disorders.

Ten years previously he had presented with incidental hypercalcemia (serum calcium 3.09 mmol/L) and hypercalciuria (24‐hour urinary calcium 10.2 mmol/day) due to primary hyperparathyroidism (serum parathyroid hormone [PTH] 7.6 pmol/L). He was successfully treated by subtotal (three‐and‐half‐gland) parathyroidectomy. Histology confirmed primary hyperparathyroidism‐related multiglandular parathyroid disease. The postoperative serum calcium (2.33 mmol/L) and PTH levels (1.5 pmol/L) normalized. There was no history of tetany or documented hypocalcemia. Genotyping for menin and RET variants were negative. Gene studies for underlying multiple endocrine neoplasia (MEN) 4, MEN5, and hyperparathyroidism‐jaw tumor were not performed. However, whole genomic sequencing of peripheral blood mononuclear cells failed to demonstrate any specified mutational abnormalities associated with genetic hyperparathyroidism syndromes (data not shown).

For the next 10 years, the patient regularly attended his local practitioner. His biochemistry remained normal (serum calcium 2.39 mmol/L) and his lumbar spine BMD measured by DXA using a lunar scanner (GE Healthcare) progressively increased from 0.92 g/cm^2^ (T‐score: −2.7) to 1.09 g/cm^2^ (T‐score: 1.1) and femoral neck BMD from 0.81 g/cm^2^ (T‐score: 2.0) to 0.83 g/cm^2^ (T‐score: 1.8).

### Laboratory investigations and bone turnover markers

Whole blood, serum, and an early‐morning 2‐hour urine void were collected after an overnight fast at presentation and again at 6 months. Samples were sent for routine biochemistry and hormonal profiles. PTH was measured using the Elecsys PTH (1–84) assay (Roche, Mannheim) and serum 1,25 dihydroxyvitamin D by the LIAISON® XL 1,25 dihydroxyvitamin D assay (DiaSorin). Serum propeptide of type 1 collagen (P1NP) was performed using an electrochemiluminescence immunoassay (Roche, Mannheim). The precision error for the assay is 4.05% for a mean of 30.7 mcg/L and 3.16% for a mean value of 200.2 mcg/L. The age‐specific reference range for a man is 16.9–42.4 ng/mL. Urinary deoxypyridinoline excretion (uDPyD) was measured using an enzyme‐linked chemiluminescent competitive immunoassay (monoclonal murine anti‐DPyD antibody) (Siemens Healthcare Diagnostics, Norwood, MA, USA). The precision error for the assay is 8% for a mean value of 98 nmol/L. The age‐specific reference range (normalized to urine creatine levels) for a man is 2.3–5.4 nmol/mmol of creatinine. Urine cross‐linked N‐terminal telopeptide (NTX) of type 1 collagen was measured by enzyme‐linked immunosorbent assay (ELISA) (NTX, Osteomark). Specimens with values exceeding 3000 nmol/mmol creatinine were diluted 1:5 using a urine diluent of known Osteomark® value (300 nmol/mmol creat) and retested. The precision error for the assay was 5% for a mean of 417 nmol/mmol creatinine and 5% for a mean value of 2640 nmol/mmol creatinine. The age‐specific reference range (normalized to urine creatine levels) for a man is 21–83 nmol/mmol of creatinine.

### Imaging and BMD

Radiographs and CT scans of the thoracolumbar spine and pelvic regions were performed to assess for the presence of vertebral fractures, lytic lesions, and skeletal abnormalities. A thoraco‐abdominal CT was used to assess for cardiorespiratory pathology. A radionuclide 99^M^ technetium‐methylene diphosphonate whole‐body bone scan combined with single‐photon emission tomography (SPEC)/CT using commercially available E.software (Siemens, USA) and a F^18^FDG PET CT using a GE Healthcare Discovery MI were used to assess skeletal activity and exclude the presence of a primary neoplasm or metastatic activity.

The BMD of the lumbar spine and hip was determined by DXA using a lunar scanner (GE Healthcare) and following the manufacturer's guidelines for patient positioning and scan acquisition. The machine's calibration is checked on a daily basis prior to each scanning session using the GE Lunar Calibration Phantom and Aluminum Spine Phantom. The precision error for the spine (L1–L4) is 1.0%, for the total femur 1.2%, and for the femoral neck 1.5%. All scans were evaluated retrospectively by the “compare” method by a single operator in a blinded fashion to eliminate bias and to ensure equivalent data acquisition.

Vertebral body and hip BMD were measured by QCT using Siemens Somatom Definition Flash CT Scanner and calculated using single‐dose dual‐energy technology, an In‐Table phantom, and QCT software. Measurements are calculated for the three lumbar spine vertebrae L2–L4 and expressed in mg/cm^3^ and for the hip in g/cm^2^, as well as the derived BMD T‐ and Z‐scores. The in vitro volumetric BMD precision was performed weekly on the scanner and calculated for the solid phantom with aqueous K_2_HPO_4_ of 0.7% at nominal density of 50–200 mg/cm^3^. The in vivo BMD precision is 2.4% for the spine at a nominal BMD of 124 mg/cm^3^ and 1.4% for the hip at a nominal BMD of 1.0 g/cm^2^. Using a digital ruler, the height, width, and depth of L2 and L3 vertebrae were determined by repeated measures on the images acquired on the QCT scout films at baseline and 6 months. The mean vertebral body bone volume was calculated to determine whether there was a significant change in the vertebral body size. Scan measurements were analyzed retrospectively and in a blinded fashion by an independent operator. The intra‐observer precision was 0.4–0.6% for height, width, and depth measurements.

### Ex vivo Micro–CT and histology

Iliac crest bone biopsy was performed using a trephine needle by a vertical approach, which enabled the assessment of subcortical and deep cancellous bone. Cortical bone could therefore not be assessed on this sample. Following fixation in paraformaldehyde, transiliac cores were scanned using a Bruker 1172 scanned at a resolution of 4.37 um using a 0.5‐mm aluminum filter, and scan settings and trabecular bone parameters were assessed using standard approaches.^(^ ^11^ ^)^ Undecalcified methylmethacrylate embedded sections were stained with toluidine blue, acid phosphatase reagents, and Goldner's trichrome to assess osteoid and tartrate‐resistant acid phosphatase (TRACP), as previously described to examine osteoclast activity.^(^ ^12^ ^)^ Dynamic bone histomorphometry to assess bone formation parameters was not performed as the patient did not receive tetracycline labeling at the time. Decalcified, paraffin‐embedded, hematoxylin and eosin (H&E)‐stained sections were examined to determine the cellular pathophysiology underlying the changes in bone mass, and polarized light was used to assess lamellar and woven bone matrix. Immunohistochemistry was performed to assess the expression of CK7, CK20, TTF‐1, Caudal type homeobox transcription factor ‐2 (CDX2) (primary and metastatic lung cancer markers), NKX3.1, SOX‐10 (metastatic prostate cancer markers), and CD117 and tryptase (mast cell markers). Fine needle aspiration of the pleural fluid was assessed for whole protein levels. The pleural fluid cytospin was assessed by a pathologist following staining for D periodic Acid Schiffs (DPAS)‐positive intracytoplasmic mucin cytokeratin ‐7, Cytokeratin8/18, and carcinoembryoinc antigen (CEA) (gastrointestinal and colorectal cancer markers), TTF1, PAX8 (thyroid and renal cancer markers), prostate‐specific antigen (PSA) (prostate cancer marker) and synaptophysin, neuron‐specific enolase (NSE), and chromogranin A (CgA) (neuroendocrine marker).

## Results

### Laboratory investigations (Table 2)

**Table 2:** Laboratory Data

Variable	Reference range^a^	Admission	6 months
Erythrocyte sedimentation rate (mm/h)	1–30	27	**17.2**
C‐reactive protein (mg/L)	0–5	**31.4**	**15.7**
Hemoglobin (g/dL)	12.8–17.5	14.8	**8.5**
White‐cell count (per μl)	4000‐11,000	4700	3400
Platelet count (per μl)	150,000‐450,000	304,000	**76,000**
Ferritin (μg/L)	30–300	667	**1740**
Protein (g/dl)			
Total	64–83	58	**47**
Albumin	36–47	35	**27**
Globulin	23–39	23	**20**
Total testosterone (nmol/L)	9–28	**7.5**	**8.3**
Insulin‐like growth factor 1 (nmol/L)	7–29	7.0	12
Calcium (mmol/L)	2.15–2.55	**1.81**	**2.04**
Phosphate (mmol/L)	0.80–1.50	1.13	1.22
Creatinine (μmol/L)	60–120	65	60
25 hydroxyvitamin D (nmol/L)	35–130	84	84
1,25 dihydroxyvitamin D (pmol/L)	60–208	**359**	No data
Parathyroid hormone (pmol/L)	1.6–6.9	**8.8**	5.2
Alkaline phosphatase (U/L)	35–110	**1852**	**849**
Bone‐specific ostase (μg/L)	5–24	**41**	**78**
Propeptide of type 1 collagen (μg/L)	13.9–85.5	**1036**	**418.3**
Urine deoxypyridinoline (nmol/mmol creat)	2.3–5.4	**19.7**	**31.9**
Urine telopeptide (nmol/mmol creat)	21–83	**3489**	No data
Urine calcium/creatinine ratio	0.2–0.4	**<0.2**	**<0.2**

^a^ Reference values are affected by many variables.

*Note*: The normative data presented are for normal adult men used at St George Hospital. Bold text represent data below or above the normal reference range.

Serum and 24‐hour urine calcium levels were reduced even after correction for low albumin, while serum PTH and 1, 25‐dihydroxyvitamin D_3_ concentrations were elevated. The normal serum 25‐hydroxyvitamin D_3_ excluded a nutritional/deprivational cause as a contributing factor of the hypocalcemia and hyperparathyroidism. Biochemical markers of bone formation (serum bone‐specific alkaline phosphatase and N‐terminal propeptide of type I procollagen) and bone resorption (urine deoxypyridinoline and N‐terminal telopeptide of type 1 collagen) were markedly raised. Full blood count, liver and kidney functions, electrolytes, immunoglobulin electrophoresis, free light chains, angiotensin converting enzyme level, serum tryptase activity, and serum fluoride levels were normal. Hepatitis B and C serology were negative and tumor markers including PSA, carbohydrate antigen (CA) 19‐9, CA 125, and CA 72‐4, carcinoembryonic antigen, and neurone‐specific enolase were in the normal range. Age‐specific serum testosterone and insulin‐like growth factor 1 levels were mildly reduced.

### Imaging studies

**Fig. 1:**
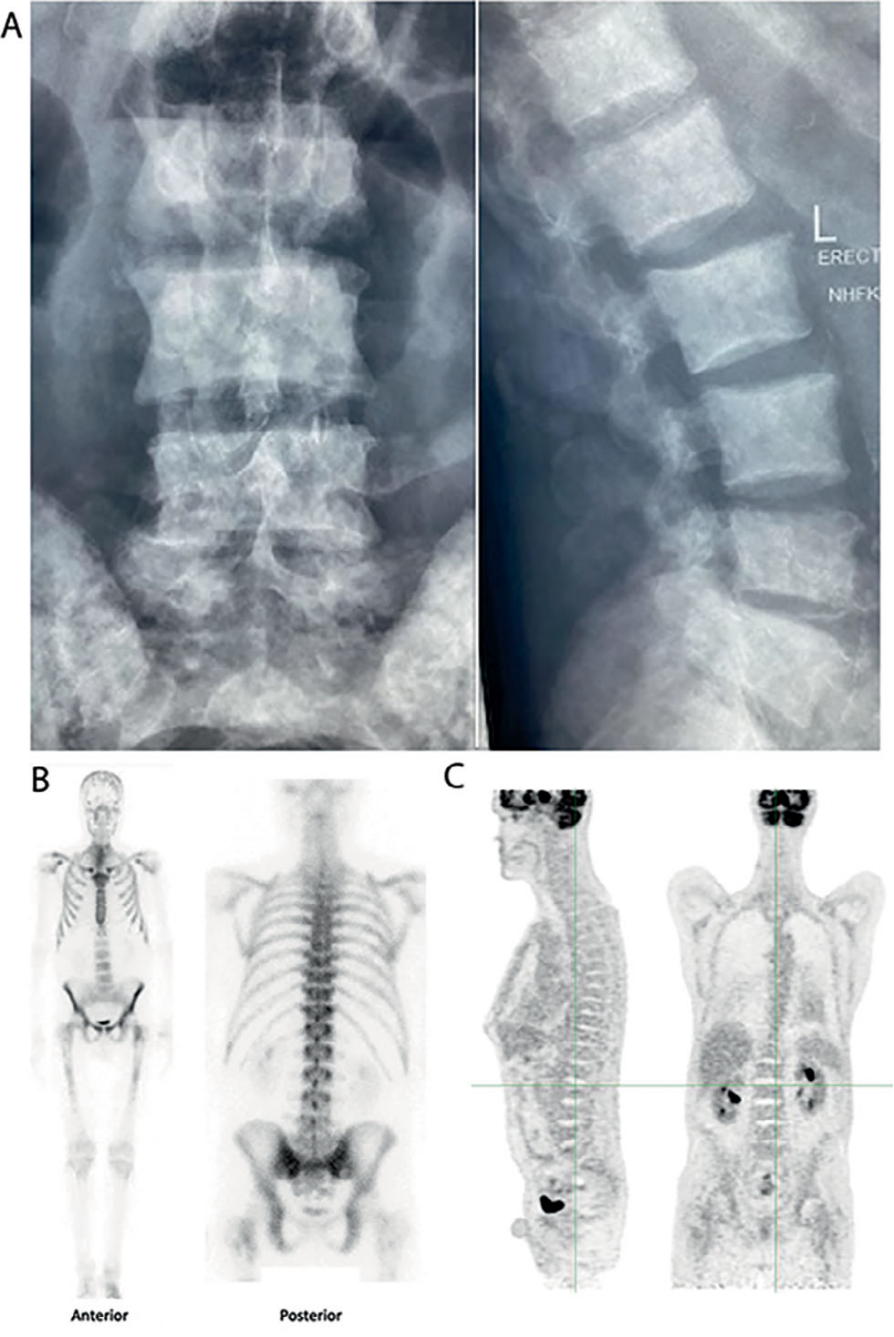
(*A*) X‐rays of lumbar spine demonstrating diffuse sclerosis with a heterogenous “mottled appearance” and no lytic lesions or fractures. (*B*) TcWBS demonstrating increased diffuse tracer uptake within skeleton compared to soft tissue. (*C*) FDG PET CT showing no focal FDG uptake to suggest a primary or secondary malignancy.

Thoracic, lumbar spine, and pelvic X‐rays (Fig. 1*A*) demonstrated diffuse sclerosis with a heterogeneous “mottled appearance” and no lytic lesions or spinal fractures. Lumbar spine CT confirmed diffuse sclerosis involving the vertebral bodies, pedicles, and spinous processes (Fig. S1). There was minimal disc and facet joint disease and no extravertebral calcification.

DXA scans were repeated and compared to scans that were performed 18 months prior to presentation. There was a striking 64% increase in the lumbar spine to 1.79 g/cm^2^ (Z‐score + 5.3) and 22% increase in the femoral neck BMD to 1.01 g/cm^2^ (Z‐score + 0.8) (see original BMD for absolute comparisons) over the time interval (Fig. S2). Lumbar spine BMD measured by QCT was markedly elevated (418.4 mg/cm^3^, Z‐score + 11.0).

CT of chest, abdomen, and pelvis demonstrated bilateral small pleural effusions measuring about 1–2 cm in depth, normal cardiac dimensions and no pulmonary infiltrate, mediastinal lymphadenopathy, hepatosplenomegaly, or intra‐abdominal tumors (Fig. S1).

Radionuclide technetium‐99 whole‐body bone scan (TcWBS) (Fig. 1*B*) confirmed diffuse increased tracer uptake throughout the whole skeleton, but most prominently in the thoracic and lumbar spine and costochondral regions (“super scan”). Renal tracer uptake was absent, compatible with increased skeletal turnover. ^18^F‐FDG PET CT (Fig. 1*C*), performed from midbrain to proximal femora, confirmed the previous CT findings of diffuse osteosclerosis and pleural effusions but no focal areas of abnormal FDG uptake in the skeleton or elsewhere to suggest an underlying primary malignancy or metastatic disease.

### Histology

#### Transiliac crest bone biopsy

**Fig. 2:**
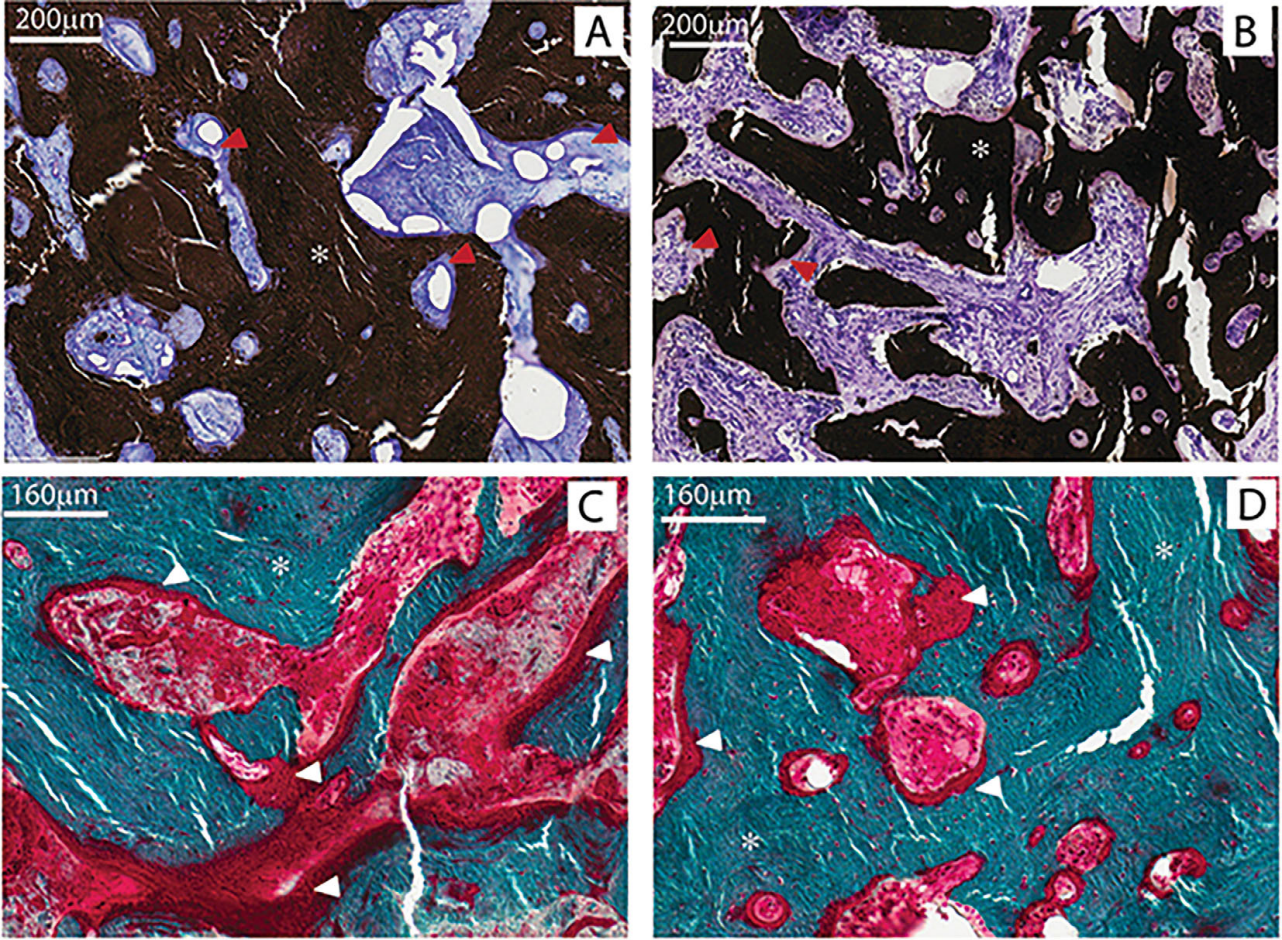
(*A, B*) Von Kossa and toluidine blue‐stained undecalcified sections of patient illiac crest biopsy showing thickened trabecular bone (black*) covered by wide osteoid seams (purple, red arrows). (*C, D*) Goldner's trichrome stained sections of patient illiac crest biopsy showing thickened trabecular bone (green matrix*) covered by wide osteoid seams (red matrix, white arrows).

**Fig. 3:**
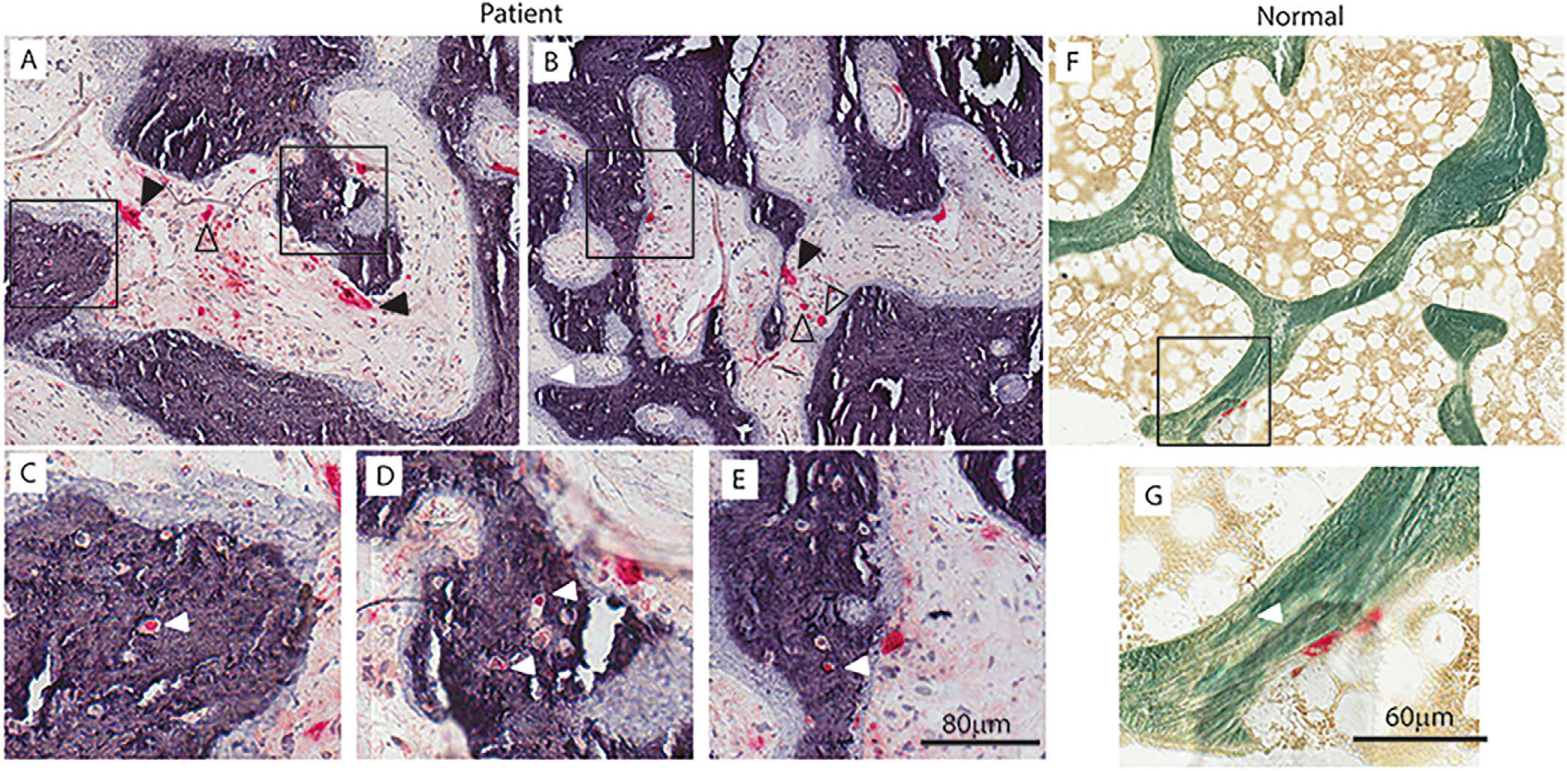
(*A–E*) TRACP‐stained images of biopsies from the patient and (*F, G*) age and sex matched normal control. (*C, D, E, G*) zoomed in images from black boxes in (*A, B, F*). Multiple large TRACP positive (TRACP^+^) cells (red) are seen on bone or osteoid surfaces (black arrows) or in the fibrous marrow space (open black arrows) in the patient. Typical rare flat TRACP^+^ cells are seen only on the bone surface in the normal biopsy. Numerous osteocyte lacunae are TRACP^+^ in the patient (white arrows *C–E*). These are not evident in the normal biopsy.

Undecalcified methyl methacrylate‐embedded, toluidine blue‐stain sections demonstrated wide thickened trabeculae composed of lamellar bone and covered by wide osteoid seams (Fig. 2). Markedly sclerotic woven bone was seen expanding into the marrow space. Staining with TRACP confirmed the presence of numerous large multinucleated giant osteoclasts within the bone marrow stroma and adjacent to bone surfaces. Prominent TRACP staining was seen in the perilacunar spaces surrounding active osteocytes within trabecular bone (Fig. 3), suggestive of osteocytic osteolysis.^(^ ^13^ ^)^ Detailed bone histomorphometry^(^ ^14^ ^)^ (Table 3) showed quantitative increases in trabecular and woven bone area, osteoid surfaces and thickness, and fibrous bone area compatible with high bone turnover. Though the osteoclast numbers were increased, the active resorption surfaces were normal. Tetracycline labeling was not performed.

**Table 3:** Bone Histomorphometry (According to ASBMR Nomenclature^(^ ^5^ ^)^)

Variable	Reference range	Patient
Trabecular bone area (%)		
Lamellar bone	21–29	46.8
Woven bone	<1%	25
Relative osteoid area (%)	1.3–3.1	4.8
Total osteoid surfaces (%)	7.1–13.9	34.8
Active resorption surfaces (%)	0.5–2.5	0.9
Total resorption surfaces (%)	2.2–6.7	4.4
Osteoclast number (cells per cm^3^)	0.1–1.3	1.4
Osteoid thickness (μM)	9.2–14.9	18.6
Fibrous area (%)	<1%	14.8

**Fig. 4:**
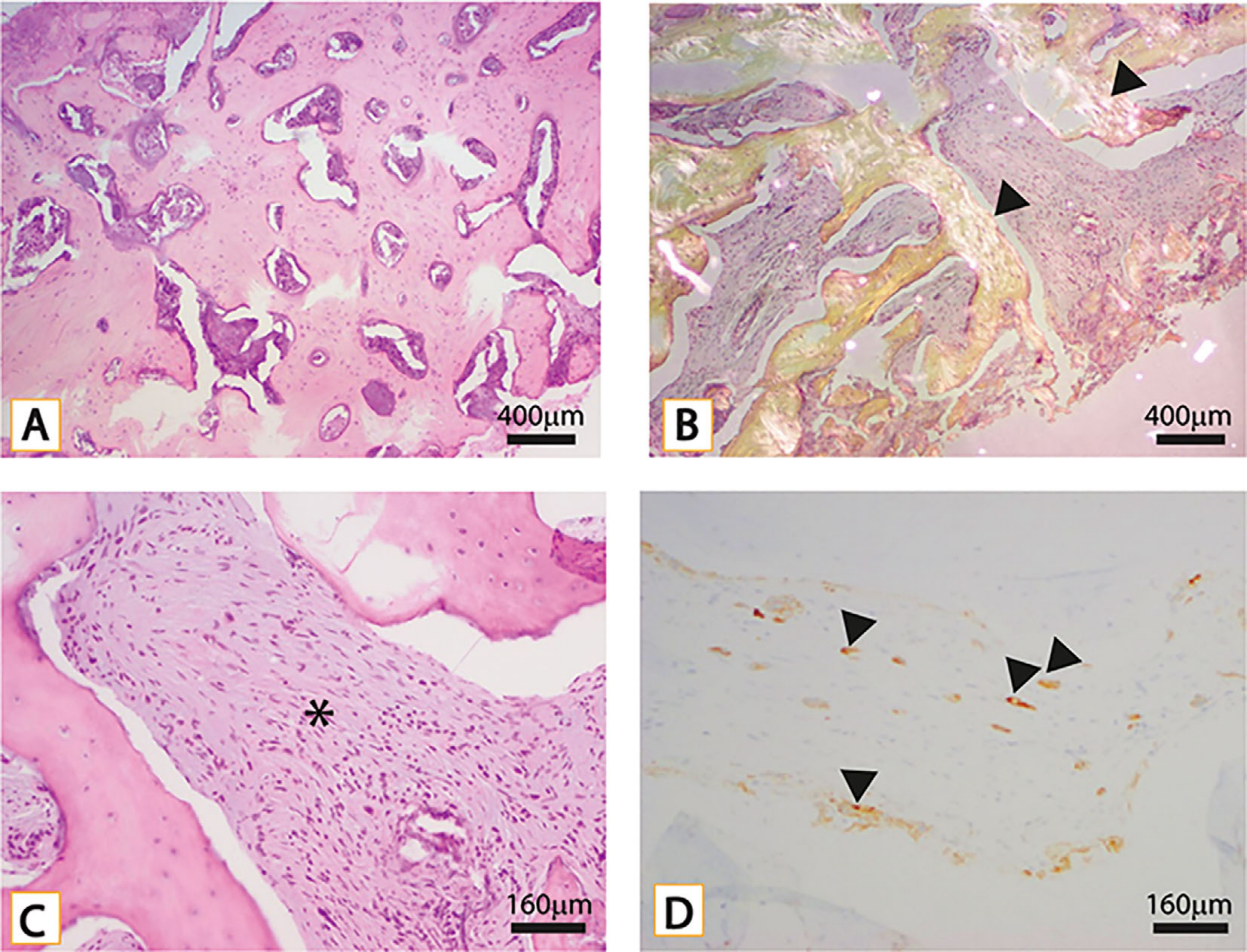
(*A*) Low‐power H&E section of bone biopsy with widespread sclerosis and intervening crushed marrow elements. (*B*) Polarized light showing woven and lamellar (black arrows) architecture. (*C*) Intermediate‐power H&E section with crushed epithelial cells in fibrous stroma (*). (*D*) Cytokeratin 7 highlighting crushed epithelial cells consistent with carcinoma (black arrows).

Decalcified, paraffin‐embedded, H&E‐stained sections showed markedly sclerotic woven and lamellar bone (Fig. 4). The marrow space was devoid of typical bone cells and adipocytes and instead filled with fibromyxoid stroma containing small clusters and single cells characterized by hyperchromatic irregular nuclei and eosinophilic cytoplasm. The tumor cells were seen diffusely infiltrating the marrow space in both cores. By immunohistochemistry, the tumor cells were positive for pancytokeratin and diffusely and strongly positive for CK7. They were negative for CK20, TTF‐1, CDX2, and a prostatic marker homeobox protein (NKX3.1). Sex determining region Y‐related high mobility group box ‐10 (SOX‐10) was negative. Mast cell marker CD117 and mast cell tryptase did not highlight an appreciable increase in mast cells. The histological features are indicative of metastatic adenocarcinoma (Fig. 4).

**Fig. 5:**
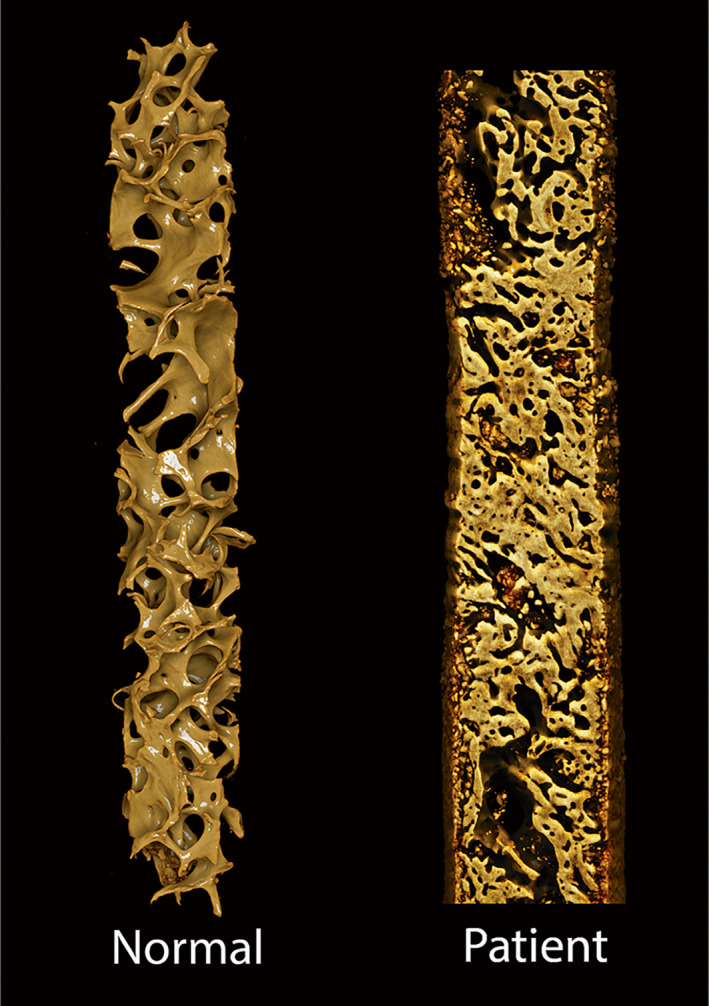
3D reconstruction of MCT scanned transiliac biopsies from a “normal” patient and the patient described herein. MCT showing a marked increase in trabecular bone volume in patient (BV/TV 55%) compared to an age‐matched male control (BV/TV 14%).

Micro‐CT^(^ ^11^ ^)^ showed qualitative changes reflecting osteosclerosis (Fig. 5). Detailed measurements (Table 4) confirmed an increase in trabecular bone volume as a ratio of the total bone volume (BV/TBV) in the patient (58%) as compared to a matched control (14%).

**Table 4:** Micro‐CT Analysis^(^ ^6^ ^)^

Variable	Reference range	Patient
Bone volume /Total volume (%)	4.8–27.9	54.90
Trabecular thickness (mm)	87–255	44.00
Trabecular separation (mm)	523–1307	76
Trabecular number (N/mm)	0.788–2.051	12.45

#### Pleural fluid cytology and skin histology

Fine needle aspiration of the pleural fluid revealed cloudy yellow fluid with an elevated total protein estimation (32 g/L) and a lactate dehydrogenase fluid/serum ratio (2.7), suggestive of an exudate (32 g/L). The pleural fluid cytospin confirmed metastatic adenocarcinoma with atypical cells showing hyperchromatic overlapping nuclei with prominent nucleoli and thickened nuclear membrane. The cell block showed cells with DPAS‐positive intracytoplasmic mucin and positive staining for CK7, CK8/18, and CEA but negative for TTF1, PAX8, PSA, synaptophysin, NSE, and CgA (Fig. S3). Excision of a skin verruca overlying the left temple bone performed under local anesthesia showed findings of a moderately differentiated adenocarcinoma similar to that found in the pleural fluid cytology.

#### Differential diagnosis

This case presented with generalized diffuse osteosclerosis, HBM, and rapid bone mass accrual. The elevated lumbar spine DXA (+5.3) and QCT Z‐scores (+11.0) confirmed a true HBM phenotype. The initial increases in BMD could be attributed to successful parathyroid surgery,^(^ ^15^ ^)^ whereas the dramatic increases seen over 18 months prior to presentation was more likely due to a different cause associated with high bone formation, increased skeletal calcium uptake, and hypocalcemia.

Two distinct genetic (sclerosing bone dysplasias and osteopetrosis)^(^ ^4^, ^5^ ^)^ and a number of acquired disorders (fluorosis, hepatitis C‐associated osteosclerosis, myelofibrosis, myeloma, lymphoma, and malignancy)^(^ ^6^, ^7^, ^8^, ^9^ ^)^ cause generalized osteosclerosis and HBM. In our patient, osteosclerosis was seen in both the axial and appendicular skeleton. Without knowledge of the prior BMD measurements, a differential diagnosis of a heterozygous sclerosing bone mass disorder could be considered. However, the clinical presentation of accelerated bone mass accrual (in 18 months) and the associated findings of adenocarcinoma in the pleural fluid, skin lesions, and bone marrow stroma favored the possibility of malignancy in the pathogenesis of the patient's bone disorder. While fluorosis, hepatitis C‐associated osteosclerosis, and hematological disorders present with a very similar pattern of bone disease, these were excluded by appropriate testing.

#### Progress

**Fig. 6:**
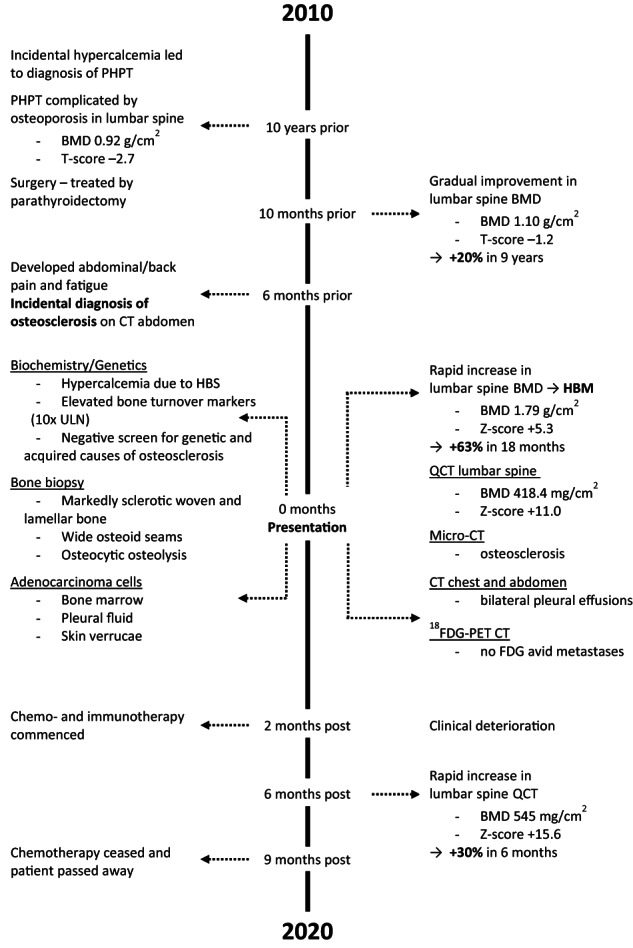
Timeline detailing sequence of events in our patient. Abbreviations: PHPT = primary hyperparathyroidism; BMD = bone mineral density; HBM = high bone mass; HBS = hungry bone syndrome; ULN = upper limit of normal; QCT = quantitative computed tomography; TV = trabecular bone volume; BV = total bone volume; ^18^FDG = 2‐deoxy‐2‐[fluorine‐18] fluoro‐D‐glucose; PET = positron emission tomography.

The patient commenced chemotherapy with carboplatin and gemcitabine and thereafter the addition of pembrolizumab for a presumed lung malignancy (no solid pulmonary tumor was ever identified on imaging modalities). At no time did he receive bone active agents. His response to chemo−/immunotherapy was poor, and his condition progressively deteriorated with recurrent pleural effusions, new onset ascites, and pancytopenia due to bone marrow failure (Table 2). His bone turnover markers remained elevated, and his BMD further increased. At 6 months after his cancer diagnosis and despite chemotherapy, lumbar spine QCT continued to increase by 30% from 418.4 mg/cm^3^ (Z‐score + 11.0) to 545 mg/cm^3^ (Z‐score + 15.6), femoral neck by 28% from 0.97 g/cm^2^ (Z‐score + 1.7) to 1.24 g/cm^2^ (Z‐score + 3.8), and total hip by 49% from 1.16 g/cm^2^ (Z‐score + 1.7) to 1.73 g/cm^2^ (T‐score + 10.8). The increases in lumbar spine BMD occurred without apparent changes in vertebral body size or volume (Fig. S3). At 9 months, the patient became very frail. Chemotherapy was withdrawn, and he was transferred to a palliative hospital for end‐of‐life care. A timeline detailing the sequence of events and progress in BMD is given in Fig. 6.

## Discussion

The severity of the osteosclerosis and HBM (QCT Z‐score + 15.6) seen in this case is exceptionally rare and estimated to occur in 1 in 10^−39^ percent of the general population.^(^ ^16^ ^)^ The extent to which the patient's BMD increased over the last 6 months of his life is unique and, to our knowledge, of such a magnitude that had never previously been reported. These increases at all skeletal sites (30%–50%) were significantly higher than those seen in *SOST*‐related sclerosing bone dysplasias^(^ ^17^ ^)^ and patients receiving anabolic agents for osteoporosis^(^ ^18^ ^)^ and greater than that seen during maximal skeletal growth from adolescence to adulthood.^(^ ^19^ ^)^

**Fig. 7:**
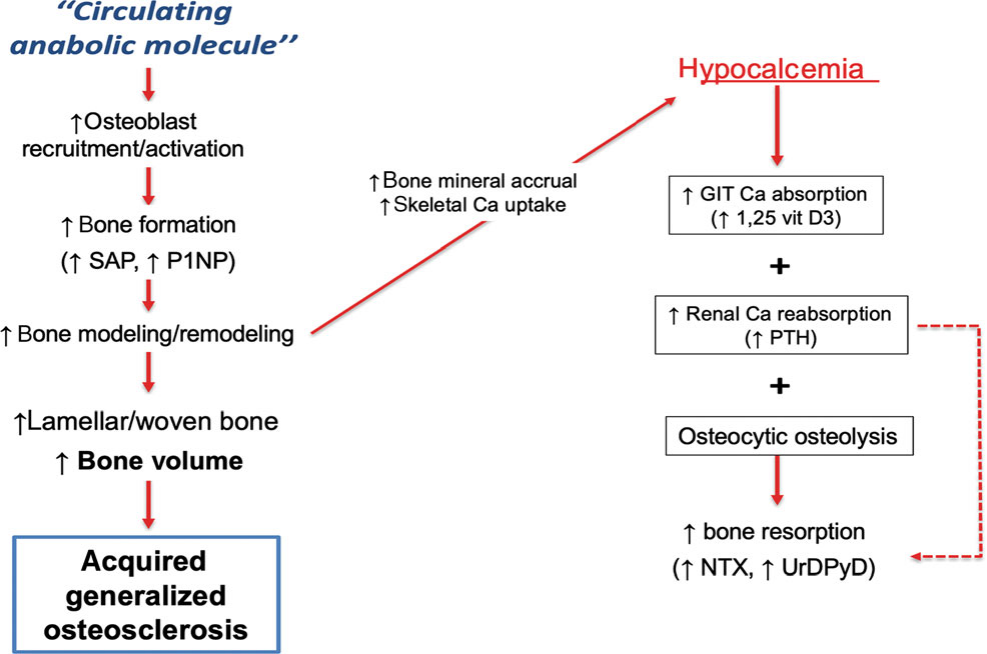
Proposed mechanism for an “anabolic paracrine molecule” causing osteoclerosis in our patient. Abbreviations: SAP = serum alkaline phosphatase; P1NP = serum procollagen type 1 N propeptide; Ca = calcium; GIT = gastrointestinal tract; 1,25 vit D3 = 1,25 dihydroxyvitamin D_3_; PTH = serum parathyroid hormone; NTX = urine telopeptide; UrDPyD = Urine deoxypyridinoline.

Numerous reports exist of histological localised sclerosis in patients with various cancers, predominantly breast and prostate, but our report is the first detailing the striking sequential changes in both DXA and QCT measurements. The presumed pathophysiological mechanisms leading to osteosclerosis in this case were complex and guessed at in Fig. 7. The increases in BMD are most likely the result of newly synthesized lamellar and woven bone by osteoblasts recruited to surfaces of multiple bone modeling units (BMUs)^(^ ^20^ ^)^ through the paracrine action of either single or multiple growth factors (there are many candidates, such as bone morphogenic proteins, insulin growth factors, fibroblast growth factors, vascular endothelium growth factors, and endothelin) secreted by circulating tumor cells in the fibro myxoma. The very high bone formation is probably a consequence of an increase in individual osteoblast activity at the cellular BMU.^(^ ^21^ ^)^ Together these mechanisms are responsible for the positive bone balance and HBM seen in this case.

Although our patient initially presented with primary hyperparathyroidism, this was surgically corrected and with no clinical recurrence at 10 years after surgery. Detailed exploration for an occult neuroendocrine tumor, as part of a MEN syndrome, and secretion of an anabolic peptide was never forthcoming. A plasma neuroendocrine hormonal screen was negative. PET/CT Ga68‐DOTATE and/or 111‐labeled somatostatin analogue scintigraphy were not performed because a primary solid tumor was never discovered. The circulating tumor cells found in the pleural fluid, ascites, and bone marrow demonstrated “classic” cytological features of adenocarcinoma. Specific immunoperoxidase staining (synaptophysin, NSE, and CgA) excluded a possible neuroendocrine tumor.

The findings of hypocalcemia and secondary hyperparathyroidism in our patient in the absence of malabsorption, vitamin D deficiency, and renal disease are thought to be a consequence of the excessive skeletal calcium demands required for the bone mass accrual. The inappropriate “tempered” PTH response may have been blunted by the previous subtotal parathyroidectomy. The rapidly expanding bone matrix requires vast amounts of calcium and phosphate crystals for adequate bone mineralization. If these needs are not met by adequate dietary calcium, gastrointestinal calcium absorption, and renal calcium conservation, hypocalcemia may occur and result in hungry bone syndrome (HBS). This condition was initially described following parathyroidectomy in patients with severe primary hyperparathyroidism^(^ ^22^, ^23^ ^)^ and, more recently, in a case of metastatic gastric carcinoma and in another individual with prostate carcinoma.^(^ ^24^ ^)^ Our patient demonstrated some of these features of HBS. Although he had evidence of hypocalcemia, this was chronic and without symptoms of tetany. The compensatory mechanisms to prevent his hypocalcemia, which included secondary hyperparathyroidism, reduced urinary calcium excretion, elevated 1,25 dihydroxyvitamin D synthesis to promote gastrointestinal calcium absorption, and osteocytic osteolysis,^(^ ^13^ ^)^ failed to restore normal calcium homeostasis. Although increases in bone formation were striking, there were also significant increases in bone resorption. There was an increase in the density of perilacunar spaces surrounding TRACP^+^ active osteocytes within the trabecular bone, indicative of osteocytic osteolysis. Unfortunately, quantitative back‐scattered electron imaging was unavailable to confirm osteocyte viability and mineralization of the osteocyte lacunar area. Osteoclast parameters were within normal ranges, but TRACP^+^ multinucleated cells were seen in increased numbers lying dormant in the bone marrow stroma and adjacent to bone surfaces (Fig. 3), most likely occurring due to the effects of circulating cytokines produced by tumor cells, as part of the well‐described vicious cycle.^(^ ^24^ ^)^

Tumor‐induced changes in osteoclast activity leading to lytic bone lesions have been thoroughly explored, and it has been revealed that lesion progression can be supressed via antiresorptive therapies.^(^ ^25^ ^)^ However, the mechanisms of osteoblastic bone metastases in cancers such as prostate and breast carcinoma are complex and less well understood.^(^ ^26^ ^)^ Sclerotic bone lesions are common in advanced prostate cancer patients and present many challenges. A number of bone anabolic pathways have been implicated in the pathology of sclerotic/osteoblastic prostate carcinoma lesions. These include bone morphogenic proteins (BMPs), osteopontin, and the Wnt/β catenin pathway, each of which stimulates downstream Runt‐domain transcription factor signaling and aberrant osteoblast activation.^(^ ^27^, ^28^ ^)^ Overactive bone formation enhances tumor growth and further compromises the bone marrow compartment. Preclinical models have demonstrated that inhibition of BMP signaling in particular can limit the osteoblastic response and, to some extent, indirectly suppress tumor growth^(^ ^27^ ^)^; however, to date these approaches have not translated to the clinic. Osteocytes have also been implicated in the regulation of bone response to tumor, but this has been less well explored due to complexities involved in assessing osteocyte function in vivo.^(^ ^29^ ^)^ Stimulation of tumor cell growth, migration, and invasion has been shown in vitro using osteocyte‐conditioned media. The simulation of mechanical stimuli in 3D osteocyte organ chips was further increased by the invasion of prostate cancer cells.^(^ ^30^, ^31^ ^)^ We were unable to find reports relating to osteoblast/osteocyte responses in patients presenting with malignant osteosclerosis, such as in this case. Nevertheless, we cannot rule out the possibility of a direct or indirect effect of malignant tumor cells, via paracrine or endocrine mechanisms, on osteoblast or osteocyte function in this patient.

Our case outlines the dynamic changes in bone homeostasis that can occur in malignancy and raises the possibility of perhaps some other novel paracrine anabolic factor (or factors) secreted by an adenocarcinoma of unknown primary that resulted in dramatic increases in BMD, HBM, and radiological osteosclerosis. An alternative explanation for our findings may be that of a novel endocrine (circulating) anabolic factor secreted by a distant occult neuroendocrine tumor. More detailed studies are planned to unravel the mystery of this patient's osteosclerosis and the mechanisms responsible for it.

## Author Contributions

**Terrence H. Diamond:** Conceptualization. **Carl Bryant:** Data curation. **Richard Quinn:** Data curation; formal analysis. **Sindhu T. Mohanty**: Generated MicroCT data and figures. **Fiona Bonar:** Formal analysis. **Paul A. Baldock**: Contributed to data interpretation and MicrCT analysis. **Michelle M. McDonald**: Contributed to conceptualization, provided specialized histology techniques and analysis, data interpretation and contributed to manuscript production.

### Peer Review

The peer review history for this article is available at https://publons.com/publon/10.1002/jbm4.10734.

## Supporting information


**Appendix S1.** Supporting information

**Figure S1.** (*A*) Lumbar spine CT: coronal views demonstrating diffuse sclerosis with a heterogenous “mottled appearance” and no lytic lesions or fractures. (*B*) Lumbar spine CT: sagittal views demonstrating diffuse sclerosis with a heterogenous “mottled appearance” and no lytic lesions or fractures. (*C*) Chest CT scan: axial views demonstrating left pleural effusion

**Figure S2.** Changes in lumbar spine and femoral neck BMD measured by DXA prior to and at 10 years after parathyroidectomy and again at 18 months when he presented with incidental osteosclerosis

**Figure S3.** (*A*) Pleural fluid H&E section of cell block with gland forming malignant cells consistent with adenocarcinoma. (*B*) Papanicolauo stain of highly atypical malignant cells with mitosis. (*C*) Periodic acid‐Schiff stain showing focal cytoplasmic positivity in keeping with adenocarcinoma. (*D*) Strong expression of CK7 in tumor cells.
