# Supplementary material for: Increased Bone Formation and Accelerated Bone Mass Accrual in a Man Presenting with Diffuse Osteosclerosis/High Bone Mass Phenotype and Adenocarcinoma of Unknown Primary
Source: JBMR Plus. 2023 Jun 14;7(8):e10734. doi: 10.1002/jbm4.10734 (PMC10443075; doi:10.1002/jbm4.10734)
Supplement: Supplementary file 1 — Appendix S1. Supporting information [file JBM4-7-e10734-s004.docx]

**Supplementary**

**Table 1.**

**QCT Data**

**Lumbar Spine BMD**

| **Vertebra BMD** | **Baseline** | **6-Months** | **% Change** |
| --- | --- | --- | --- |
| L2 – mg/cm^3^ | 403 | 545 | +35 |
| L3 – mg/cm^3^ | 464 | 609 | +31 |
| L4 – mg/cm^3^ | 388 | 481 | +24 |
| Mean L2-4 – mg/cm^3^  (Z-Score) | 418.4  (+11.0) | 545  (+15.6) | +30 |

**Lumbar Spine Dimensions**

| **Vertebra** | **Baseline** | **6-Months** | **% Change** |
| --- | --- | --- | --- |
| L2 – Height (mm) | 31.3 | 31 |  |
| Width (mm) | 50.9 | 53 |  |
| Depth (mm) | 40.7 | 42 |  |
| Volume (cm^3^) | 64.8 | 69.0 | *+6* |
|  |  |  |  |
| L3 – Height (mm) | 30.7 | 31.4 |  |
| Width (mm) | 54.7 | 50.2 |  |
| Depth (mm) | 39.8 | 40 |  |
| Volume (cm^3^) | 66.8 | 63.1 | *-6* |
|  |  |  |  |
| L2-3 Volume (cm^3^) | 65.8 | 66.0 | +0.2 |

**Hip BMD**

| **Femur** | **Baseline** | **6-Months** | **% Change** |
| --- | --- | --- | --- |
| Femoral Neck |  |  |  |
| g/cm^2^  mg/cm^3^  Z-score | 0.97  280  +1.7 | 1.24  346  +3.8 | +28  +24 |
| Total Hip |  |  |  |
| g/cm^2^  mg/cm^3^  Z-score | 1.16  404  +5.3 | 1.73  577  +10.8 | +49  +43 |

**SUPPLEMENTARY LEGENDS**

Supplementary **Figure 1.**

1. Lumbar spine CT: coronal views demonstrating diffuse sclerosis with a heterogenous ‘mottled appearance’ and no lytic lesions or fractures.
2. Lumbar spine CT: sagittal views demonstrating diffuse sclerosis with a heterogenous ‘mottled appearance’ and no lytic lesions or fractures.
3. Chest CT scan: axial views demonstrating left pleural effusion

Supplementary **Figure 2**.

Changes in lumbar spine and femoral neck BMD measured by DXA prior to and at 10-years after parathyroidectomy and again at 18-month when he presented with incidental osteosclerosis.

Supplementary **Figure 3**.

Pleural fluid cytology.

1. Haematoxylin and Eosin section of cell block with gland forming malignant cells consistent with adenocarcinoma.
2. Papanicolaou stain of highly atypical malignant cells with mitosis.
3. Periodic acid–Schiff stain showing focal cytoplasmic positivity in keeping with

adenocarcinoma.

1. Strong expression of Cytokeratin 7 in tumour cells
