# Supplementary figures and images for: Increased Bone Formation and Accelerated Bone Mass Accrual in a Man Presenting with Diffuse Osteosclerosis/High Bone Mass Phenotype and Adenocarcinoma of Unknown Primary
Source: JBMR Plus. 2023 Jun 14;7(8):e10734. doi: 10.1002/jbm4.10734 (PMC10443075; doi:10.1002/jbm4.10734)

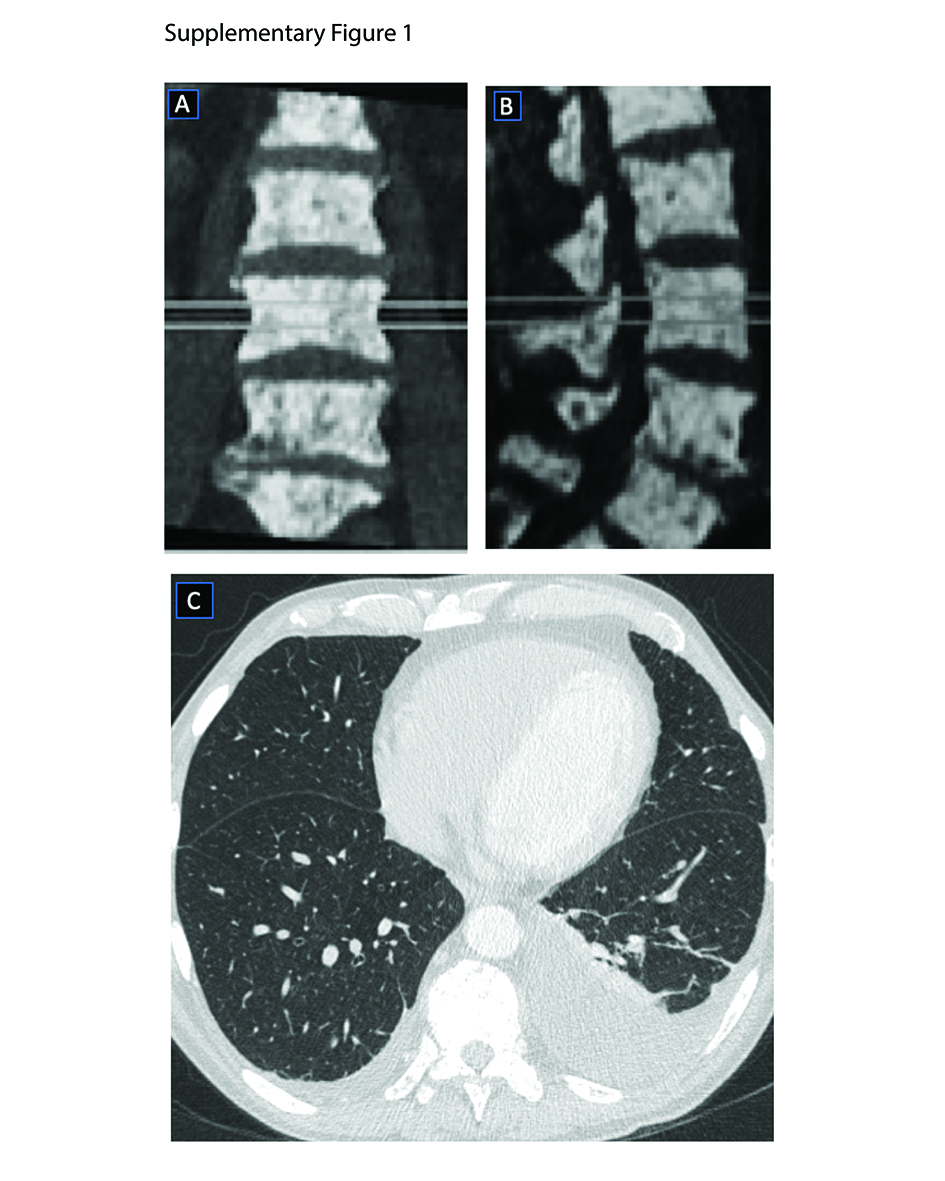

Supplement: Supplementary file 2 — Figure S1. (A) Lumbar spine CT: coronal views demonstrating diffuse sclerosis with a heterogenous “mottled appearance” and no lytic lesions or fractures. (B) Lumbar spine CT: sagittal views demonstrating diffuse sclerosis with a heterogenous “mottled appearance” and no lytic lesions or fractures. (C) Chest CT scan: axial views demonstrating left pleural effusion [file JBM4-7-e10734-s002.tif]

Supplementary Figure 2

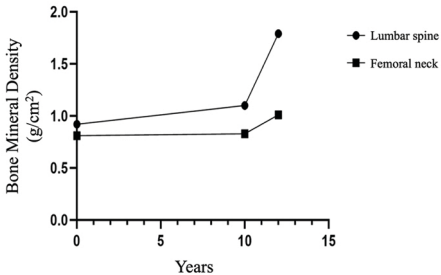

Supplement: Supplementary file 3 — Figure S2. Changes in lumbar spine and femoral neck BMD measured by DXA prior to and at 10 years after parathyroidectomy and again at 18 months when he presented with incidental osteosclerosis [file JBM4-7-e10734-s001.pdf]

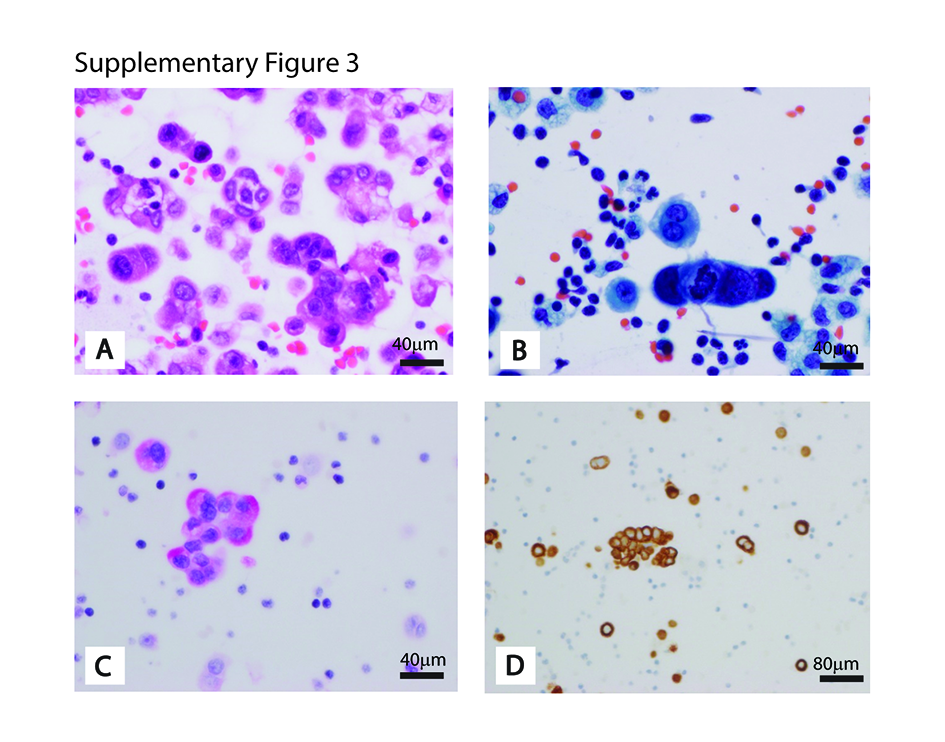

Supplement: Supplementary file 4 — Figure S3. (A) Pleural fluid H&E section of cell block with gland forming malignant cells consistent with adenocarcinoma. (B) Papanicolauo stain of highly atypical malignant cells with mitosis. (C) Periodic acid‐Schiff stain showing focal cytoplasmic positivity in keeping with adenocarcinoma. (D) Strong expression of CK7 in tumor cells. [file JBM4-7-e10734-s003.tif]
